# Amino acid metabolism-based molecular classification of colon adenocarcinoma*via in silico* analysis

**DOI:** 10.3389/fimmu.2022.1018334

**Published:** 2022-10-20

**Authors:** Yile Xie, Huimin Chen, Jing-Yuan Fang

**Affiliations:** State Key Laboratory for Oncogenes and Related Genes, Key Laboratory of Gastroenterology and Hepatology, Ministry of Health, NHC Key Laboratory of Digestive Diseases, Division of Gastroenterology and Hepatology, Shanghai Institute of Digestive Disease, Renji Hospital, School of Medicine, Shanghai Jiao Tong University, Shanghai, China

**Keywords:** amino acid metabolism, colon adenocarcinoma, classification, prognosis, immune signature, therapy

## Abstract

Amino acid metabolism is closely related to the occurrence and development of colon adenocarcinoma (COAD). Studies on the relationship between COAD and the expression of amino acid metabolism are still rare. Based on *in silico* analysis, we used 358 amino acid metabolism-related genes (AAMRGs) to determine the amino acid metabolism characteristics and then classified COAD into two distinct subtypes, namely AA1 and AA2. Then we analyzed the clinical characteristics, somatic mutation landscape, transcriptome profile, metabolism signatures, immune infiltration, and therapy sensitivity of these two subtypes. The AA1 subtype had inferior overall survival and was characterized by lower amino acid metabolic activity, higher tumor mutation burden, and higher immune cell infiltration, while AA2 displayed higher metabolic activity and relatively better survival. Furthermore, the AA1 subtype was likely to benefit from irinotecan in chemotherapy and immune checkpoint blockade therapy including programmed cell death protein-1 (PD-1) and cytotoxic T-lymphocyte-associated protein-4 (CTLA-4) immune checkpoint inhibitor but was resistant to targeted therapy cetuximab. The AA2 subtype showed higher sensitivity to 5-fluorouracil and oxaliplatin. To provide perspectives on cell-specific metabolism for further investigation, we explored metabolic activity in different cell types including lymphocytes, mast cells, myeloid cells stromal cells, and epithelial cells *via* colorectal cancer single-cell data. Additionally, to assist in clinical decision-making and prognosis prediction, a 60-AAMRG-based classifier was generated and validated in an independent cohort.

## Introduction

Colorectal cancer is the third most common new case of cancer and the second most common cause of cancer death in 2020 (1). Colon adenocarcinoma (COAD) accounts for 69.7% of all colorectal cancer (2). Due to the high heterogeneity, COAD brings more challenges to clinical treatment and management. The heterogeneity derives from many aspects, including genetic and epigenetic alterations, tumor microenvironment (TME) cell population diversity, microbiome multiformity, and metabolism adaptations. The treatment methods for COAD are mainly surgery, chemotherapy, targeted therapy, and immunotherapy and the therapeutic response is also influenced by diverse factors. Therefore, it is necessary to have a deeper understanding of the biodiversity of COAD, especially its relationship with clinical characteristics. For instance, the previous classical gene expression-based classification consensus molecular subtypes (CMSs) captured the intrinsic heterogeneity of colorectal cancer, in which colorectal cancer could be classified into 4 distinguishing types, CMS1 (immunotype), CMS2 (classical type), CMS3 (metabolic type), and CMS4 (mesenchymal type) (3). In addition, some research on COAD molecular typing and prediction models based on transcriptome data have emerged, which provide new aspects for accurate classification and treatment effect prediction of COAD (4, 5).

Metabolic reprogramming is a major feature of tumors and is involved in rapid growth, evasion of immune clearance, and adaptation to the metastatic environment (6). There has been some thorough and detailed research focused on the role of glucose metabolism, lipid metabolism, and amino acid metabolism in cancers (6, 7). Amino acid metabolism has extremely extensive effects in producing materials for metabolite biosynthesis, epigenetic modification, bioenergy supply, detoxifying ammonia, and maintaining intracellular redox status (8). Emerging studies have pointed out the significant participatory role of specific amino acid metabolisms, such as glutamine (9, 10), tryptophan (11), cystine (12), and serine (13), in COAD progression and resistance to various therapies. Amino acid-related metabolic genes may serve as a prognostic prediction model for COAD. In addition, targeting amino acid metabolism may reshape the immune microenvironment, overcome immunotherapy resistance, and improve the efficacy of existing treatments (7). A growing number of metabolism-related molecular models have been applied to COAD classification (14–16); however, the amino acid metabolism-related gene expression-based classifier has not been reported.

Recognizing that the high-throughput transcriptomics mirrors numerous molecular features behind tumor phenotype and clinical behavior, we envisioned that classifying COAD tumors based on amino acid metabolism will deepen our understanding of the metabolic heterogeneity of COAD from a new perspective and contribute to precise therapy. In this study, we aim to divide primary COAD samples into subgroups based on curated amino acid metabolism-related genes (AAMRGs), and evaluate the clinical variables, molecular features, potential therapy response through *in silico* bioinformatic analysis, then establish a classifier assisting clinical decision-making and prognosis prediction.

## Materials and methods

### Patient and sample data collection

COAD transcriptome data for classification were accessed from The Cancer Genome Atlas (TCGA), and 420 primary COAD patients with transcriptome data with corresponding overall survival information extracted from the Genomic Data Commons (GDC) Data Portal (https://portal.gdc.cancer.gov/) were enrolled in this study for *in silico* analysis. Patients with an overall survival time of fewer than 30 days were removed to prevent bias. The gene expression value was transformed into log2 [transcripts per kilobase million (TPM) +1] for further analysis. Missing values in clinical information were excluded when comparing differences in clinical characteristics between subtypes. Gene somatic mutation data (MAF files) were available in 372 of the above 420 patients in TCGA-COAD datasets and were also obtained *via* the GDC Data Portal.

### Colon adenocarcinoma subtypes based on amino acid-related genes

For further *in silico* analysis, a total of 360 amino acid metabolism-related genes (AAMRGs) were acquired from previous work **(** **Table S1** **)** (17, 18), and 358 of them were detected in TCGA-COAD transcriptome data. Then, we performed K-means consensus clustering with the gene expression profiles of 358 AAMRGs to identify subtypes by using the CancerSubtypes package in R software (19). The following details were set for subtyping: number of repetitions = 1,000 bootstraps; pItem = 0.8 (resampling 80% of any sample); maxK=10 (k-means clustering with up to 10 clusters). An appropriate number of clusters was determined based on the clustering results and clinical ease of use. The Kaplan−Meier method with a log-rank test was performed to compare overall survival differences between the subtypes.

### Characteristics specific to the AA subtypes

To investigate multiple characteristics between AA subtypes, we compared clinical and molecular features between the two AA subtypes. A Chi-square test was used to explore the clinical feature distribution between different AA subtypes. The somatic mutation profile of COAD patients from TCGA was analyzed with the maftools R package (20). Gene expression profiles were also utilized for calculating the distance of samples from four CMSs *via* the CMScaller R package (21). Transcriptomic alterations were compared between AA subtypes with differentially expressed gene analysis *via* the limma R package (22)**(** **Table S2** **)**. Pathway enrichment analysis of gene ontology biological progress (GOBP) was performed based on differentially expressed genes (P value <0.05 and log2FC >0.5), and gene set enrichment analysis (GSEA) based on the Reactome pathway database was carried out with genes with statistical significance *via* cluster profile (23) and the ReactomePA R package (24).

To evaluate different biological process activities based on gene expression profiles, single-sample gene set enrichment analysis (ssGSEA) was performed to compute enrichment scores *via* the GSVA R package (25), and 114 metabolism-related pathways (26) and 5 immune-related signatures, including anti-CTLA-4 resistance-associated MAGE-A (CRMA) (27), IFN-gamma response (28), immune checkpoint (29), hot tumor (30) and EGFR signatures (31), were collected for ssGSEA. Pearson correlation analysis was applied to investigate the correlation between amino acid metabolism-related genes and immune-related signature enrichment scores.

### Estimation of immune cell infiltration

The CIBERSORT algorithm was used to estimate the infiltration of a total of 22 immune cell populations in COAD samples by using their cell-specific gene signatures (32). Additionally, the Microenvironment Cell Populations-counter (MCP-counter) method was performed to evaluate the abundance of eight immune and two nonimmune stromal cell populations *via* the MCPcounter R package (33), and the ESTIMATE algorithm was applied for estimating immune and stromal fractions *via* the estimate R package (34).

### Generation and validation of the AA classifier

The top 30 significantly differentially expressed AAMRGs with the largest log2FC value in both AA subtypes were selected for the development of the prediction model, and thus, a 60-gene classifier was generated **(** **Table S3** **)**. For validation, the 60-gene classifier was carried out on the TCGA-COAD dataset itself and an independent dataset GSE41258 from the Gene Expression Omnibus (GEO, http://www.ncbi.nlm.nih.gov/geo/) database based on the Nearest Template Prediction (NTP) classification, a method that allows us to apply given signatures to individual sample class prediction on GenePattern (https://www.genepattern.org/). Overall survival analyses were also performed by Kaplan−Meier methods and compared by the log-rank test. The concordance correlation coefficient between the AA subtypes and predicted AA subtypes in the TCGA cohort was calculated *via* the DescTools R package (35).

### Estimation of the potential therapy response

To predict the response to three first-line treatment drugs (5-fluorouracil, oxaliplatin, and irinotecan) between the two AA subtypes, the oncoPredict R package (36) was implemented using ridge regression to estimate the half-maximal inhibitory concentration (IC50) for each COAD sample based on the gene expression profile from the Genomics of Drug Sensitivity in Cancer 2.0 (GDSC2) database (37). All parameters were set to recommended values.

To predict target therapy (cetuximab and bevacizumab) and immunotherapy (CTLA4 and PD1 monoclonal antibody) response between AA subtypes, SubMap analysis on GenePattern was applied for comparing the expression data between AA subsets with colorectal cancer patients treated with cetuximab (GSE5851) or bevacizumab (GSE53127) and melanoma patients treated with immunotherapies extracted from previous work (38).

### Estimation of cell type-specific metabolic activities based on single-cell data

Colorectal single-cell RNA sequence datasets GSE144735 were downloaded from the GEO database. The VISION algorithm was used to score each cell type in metabolic pathways obtained from KEGG and REACTOME *via* the scMetabolism R package (39).

### Statistical analyses

Analyses were performed based on R program (V.4.1.1). Overall survival analyses were performed by Kaplan−Meier methods and compared by the log-rank test. A Chi-square test was carried out to compare clinical characteristics, gene mutation rates, and therapy response ratios between AA subtypes. Pearson correlation analysis was applied to investigate the correlation between amino acid metabolism-related genes and immune-related signature enrichment scores. The Wilcoxon test was used to compare the means of the two groups. P value < 0.05 was considered statistically significant.

## Results

### Consensus clustering identifies two metabolism subtypes in COAD

**Figure 1:**
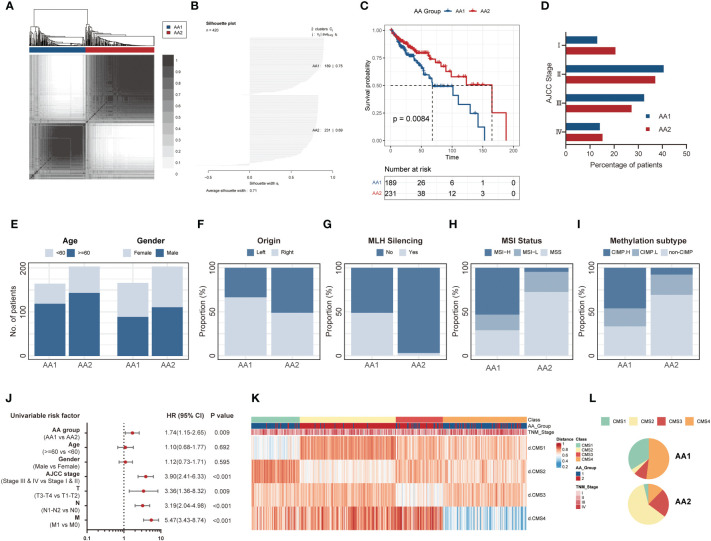
K-means consensus clustering identifies two metabolism subtypes in COAD. **(A)** Consensus clustering matrix heatmap and dendrogram for k=2 shows two distinct group patients based on the amino acid metabolism genes in the TCGA dataset. **(B)** Plot for silhouette width of 420 TCGA primary COAD patients indicating each object of the classification fit into respective cluster well. **(C)** Overall survival of two AA subtypes in the TCGA cohort. (Log-rank test). **(D)** The percentage of patients with AJCC staging between AA subtypes. **(E–I)** The proportion of clinical characteristics including age and gender **(E)**, primary tumor location **(F)**, MLH silence rate **(G)**, MSI status **(H)**, and methylation subtype **(I)** between two AA subtypes. (Chi-square test). **(J)** Univariate analysis of AA group, age, gender, AJCC stage, and TNM stage in overall survival. Univariate Cox Proportional Model was used to estimate the hazard ratios. **(K, L)** The distribution of CMS subtypes in AA subtypes. COAD: colon adenocarcinoma, MLH: MutL Homolog 1, MSI: Microsatellite instability, CIMP, CpG island methylator phenotype; AJCC, American Joint Committee on Cancer; HR, hazard ratio; CMS, consensus molecular subtypes.

Based on 358 detected genes of 360 previously reported amino acid metabolism-related genes (17, 18) (**Table S1**), we carried out K-means consensus clustering on transcriptome data comprising 420 primary COAD patient samples from TCGA and divided them into two subtypes, AA1, and AA2. The overall survival of these two subtypes was significantly differentiated, and the AA1 subtype displayed a worse prognosis (**Figures 1A–C**). For further exploration, we investigated the clinicopathological features of these two subtypes. There were no significant differences in age, gender, or American Joint Committee on Cancer (AJCC) stage between the two subtypes (**Figures 1D, E**). We observed that the AA1 phenotype could be described as a COAD subtype predominantly originating from the right colon and was associated with a higher MutL Homolog 1 (MLH1) silent mutation rate, a higher proportion of microsatellite instability (MSI) status, and a CpG island methylator phenotype (CIMP) subtype compared to the AA2 subtype (**Figures 1F–I**). Univariate analysis demonstrated that the AA subtype was an independent predictor for clinical prognosis (**Figure 1J**). Furthermore, using CMS classification (3), samples of the AA1 subtype were mainly classified into CMS1 and CMS4, the worst prognosis subtype in CMSs, while samples of AA2 were mainly classified into CMS2 and CMS3 with relatively better survival (**Figures 1K, L**). These results suggest that the heterogeneity in amino acid metabolism may be connected with the prognosis of COAD.

### Correlation of the AA subtypes with tumor mutations

**Figure 2:**
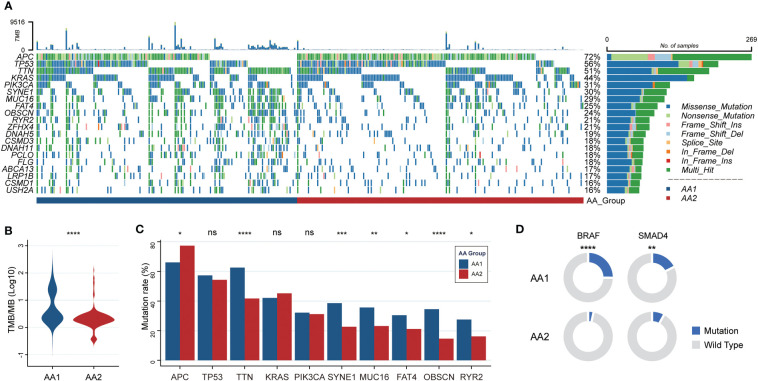
Correlation of the AA subtypes with tumor mutations. **(A)** OncoPrint of the mutation landscape of top 10 genes in AA1 and AA2 subtypes. **(B)** Tumor mutation burden between two subtypes. (Mann-Whitney Wilcoxon test) **(C)** The mutation rates of the most frequent mutant genes in two subtypes. (Chi-square test) **(D)** The mutation rates of key driver genes *BRAF* and *SMAD4* in two subtypes. (Chi-square test). *P <0.05, **P < 0.01, ***P < 0.001, ****P < 0.0001, n.s. , not significant.

The fact that AA1 tumors were intensively associated with hypermutation status and highly consistent with hypermutated CMS1 subtypes drove us to investigate whether there were mutation landscape differences between the two subtypes. The somatic mutation panorama showed that the overall mutation rate in AA1 was higher **(** **Figure 2A** **)**. Consistent with the clue offered by MSI status and CMS classification, the tumor mutation burden (TMB) was significantly higher in the AA1 subtype than in the AA2 subtype (**Figure 2B**). *APC, TP53, TTN, KRAS, PIK3CA, SYNE1, MUC16, FAT4, OBSCN,* and *RYR2* were the top 10 mutant genes in two subgroups, among which *TTN*, *SYNE1*, *MUC16*, *OBSCN*, and *FAT4* acquired higher mutation rates in the AA1 subtype while APC acquired higher mutation rates in AA2 subtype (**Figure 2C**). In addition to those high-frequency mutations, we examined the mutation rates of several driver genes reported previously (40), *BRAF* (41)*, SMAD4* (42), and *EGFR* (43, 44), and found that AA1 displayed higher *BRAF* and *SMAD4* mutation rates (**Figure 2D**).

### Correlation of the AA subtypes with transcriptome differences

**Figure 3:**
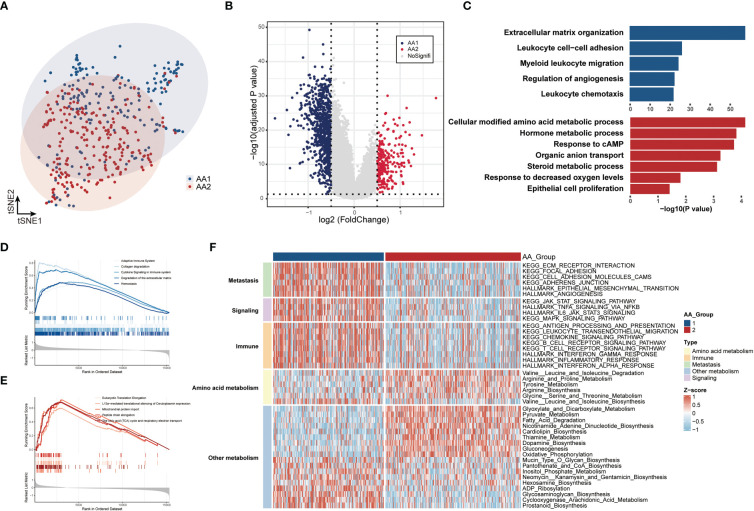
Correlation of the AA subtypes with transcriptome differences. **(A)** T-SNE analysis of transcriptome data from two subtypes. **(B)** Volcano plot of the differential expressed genes between two subtypes. **(C)** Gene Ontology Biologic Process of the top differentiated pathways between two subtypes. **(D, E)** ssGSEA of the enriched pathways in two subtypes. **(F)** Heatmaps of the signaling, immune, metastasis, and metabolism-related signatures in two subtypes.

To better characterize the features of the two subtypes, we performed transcriptomic data differential analysis. The gene expression pattern differed significantly between AA1 and AA2 (**Figures 3A, B**) (**Table S2** **)**. Pathway enrichment analysis based on gene ontology biological process (GOBP) showed that AA1 enriched pathways were mainly focused on ‘extracellular matrix organization’, ‘immune cell chemotaxis and migration’, and ‘adaptive immune’, while AA2 enriched pathways in energy metabolism such as ‘amino acid, hormone, steroid metabolic process’, and ‘cell proliferation’ (**Figure 3C**). Gene set enrichment analysis (GSEA) based on the Reactome pathway database drew a similar conclusion: AA1 was enriched with extracellular matrix remodeling and immune pathways, while AA2 was closely related to metabolism-related events. Furthermore, we analyzed the activation status of pathways involved in various biological processes, including signaling, immunity, metastasis, and metabolism, between the two subtypes through ssGSEA **(** **Figure 3F** **)**. The AA1 subtype was also active in several colon cancer progression-related signaling, immunity, and metastasis pathways such as epithelial-mesenchymal transition, angiogenesis, JAK-STAT signaling, and inflammatory pathways (**Figure 3F**). In amino acid metabolism, AA1 was only activated in ‘valine, leucine and isoleucine biosynthesis pathway’, while AA2 had an extensive activation in ‘arginine and proline metabolism’, ‘tyrosine metabolism’, ‘glycine, serine and threonine metabolism’ pathways. In addition, among other metabolism pathways, we found several inflammatory pathways, including ‘ADP ribosylation’, ‘glycosaminoglycan biosynthesis’, ‘prostanoid biosynthesis’, and ‘cyclooxygenase arachidonic acid metabolism’, were significantly activated in AA1. While energy metabolism-related pathways such as ‘oxidative phosphorylation’, ‘gluconeogenesis’, and ‘fatty acid degradation’ were highly activated (**Figure 3F**).

### Correlation of the AA subtypes with immune infiltration

**Figure 4:**
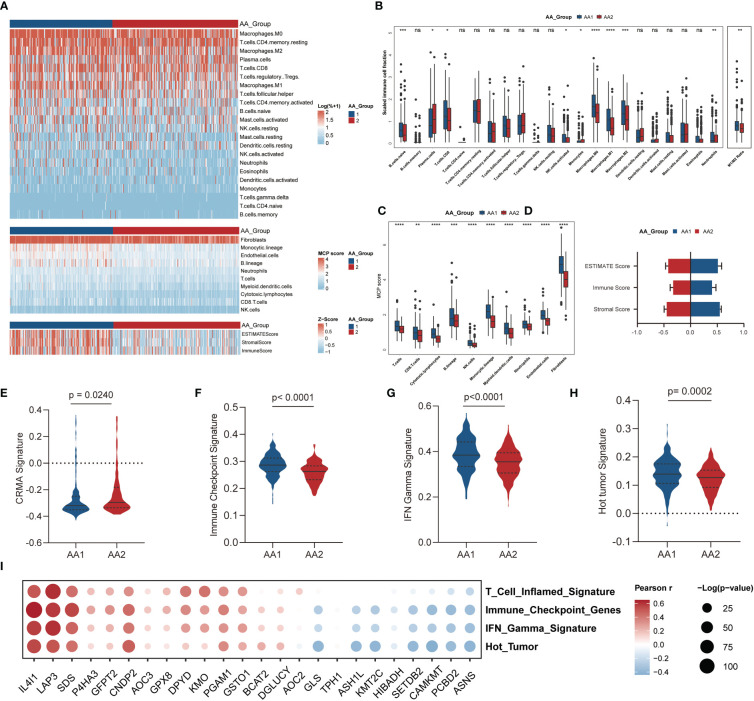
Correlation of the AA subtypes with immune infiltration. **(A)** Heatmap of the abundance of tumor microenvironment cell fractions using CIBERSORT, MCP-counter, and ESTIMATE algorithm between two subtypes. **(B)** Boxplot of the abundance of immune cell populations distinguished by two subtypes analyzed by the CIBERSORT algorithm. (Mann-Whitney Wilcoxon test) **(C)** Boxplot of the abundance of immune cell populations, fibroblasts, and endothelial cells distinguished by two subtypes analyzed by MCP-counter algorithm. (Mann-Whitney Wilcoxon test) **(D)** Boxplot of ESTIMATE score, immune score, and stromal score in two subtypes analyzed by ESTIMATE algorithm. (Mann-Whitney Wilcoxon test) **(E–H)** Tumor immune-related signatures in two subtypes. (Mann-Whitney Wilcoxon test) **(I)** Bubble chart of the correlation of main AAMRG with immune scores. *P <0.05, **P < 0.01, ***P < 0.001, ****P < 0.0001, n.s. , not significant.

The evolution of cancer is strongly dependent on the complicated tumor microenvironment (TME), which comprises a variety of cell types, including fibroblasts, endothelial cells, and immune cells. Given the distinct differences in immune pathway characteristics in the two subtypes **(** **Figures 3C–F** **)**, we investigated the immune microenvironment features through three algorithms: CIBERSORT (32), Microenvironment Cell Populations-counter (MCP-counter) (33), and ESTIMATE (34) (**Figure 4A**). *Via* the CIBERSORT algorithm, 22 immune cell fractions were calculated in each sample, and the fractions of naive B cells, CD8 T cells, activated NK cells, monocytes, macrophages (M0, M1, M2), and neutrophils were significantly higher in the AA1 subtype, while plasma cells were higher in the AA2 subtype (**Figure 4B**). Notably, the M1/M2 macrophage ratio was higher in AA1 (**Figure 4B**). The MCP-counter results showed that all evaluated types of immune cells and mesenchymal cells (endothelial cells and fibroblasts) were significantly enriched in AA1 than in AA2 (**Figure 4C**). The immune score and stromal score of the ESTIMATE algorithm resembled the conclusions of the previous two algorithms (**Figure 4D**).

We observed that the CRMA score was higher in the AA2 subtype **(** **Figure 4E** **)**, which suggested that the AA2 subclass may be linked with primary resistance to anti-CTLA-4 therapy. Other immune-related signatures, such as IFN gamma (28), immune checkpoint (29), and hot tumor (30) were higher in AA1 (**Figures 4F-H**), which indicated that AA1 may benefit more from immunotherapy. Correlation analysis of immune-related signatures with differential amino acid metabolism-related genes showed that *IL4I1, LAP3, and SDS* were the top three genes with the highest positive correlation, while *ASNS, PCBD2*, and *CAMKMT* were negatively correlated with immune signatures (**Figure 4I**).

### Correlation of the AA subtypes with treatment prognosis

**Figure 5:**
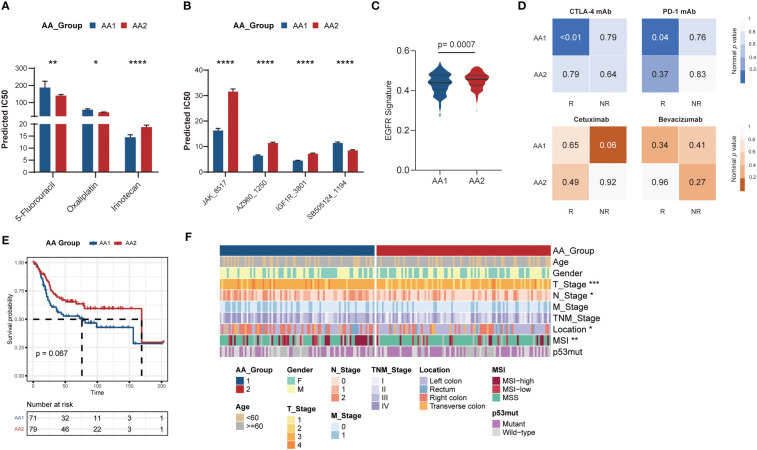
Correlation of the AA subtypes with treatment prognosis. Generation and validation of the AA classifier in the GEO dataset. **(A)** Bar chart of the predicted IC50 of COAD chemotherapy drugs 5-fluorouracil, oxaliplatin, and irinotecan of two AA subtypes. (Mann-Whitney Wilcoxon test). **(B)** Bar chart of the predicted IC50 of JAK inhibitor (JAK_8517 and AZ960_1250), IGF1R inhibitor (IGF1R_3801), and TGFβ/SMAD4 receptor inhibitor (SB505124_1194) of two AA subtypes. (Mann-Whitney Wilcoxon test) **(C)** EGFR signatures in two subtypes. (Mann-Whitney Wilcoxon test) **(D)** SubMap analysis for immunotherapy prediction in melanoma cohort (upper) and the response of targeted therapy cetuximab and bevacizumab in colorectal cancer cohort (bottom). **(E)** Overall survival of two AA subtypes typed by 60-gene classifier in GSE41258 cohort. (Log-rank test) **(F)** Clinical and pathological characteristics of the two subtypes in the GSE41258 cohort. *P <0.05, **P < 0.01, ***P < 0.001, ****P < 0.0001.

According to the aforementioned widely differed molecular characteristics between the AA1 and AA2 subtypes, we hypothesized that the two subtypes might also benefit differently from chemotherapy, immunotherapy, or targeted therapy. We predicted the half maximal inhibitory concentration (IC50) of three first-line chemotherapy drugs of patients in the AA1 and AA2 subtypes based on the GDSC2 database and found that AA2 was more sensitive to 5-fluorouracil and oxaliplatin, while AA1 was more sensitive to irinotecan (**Figure 5A**), which helped us to provide a rational drug application for AA subtypes. In addition, we found that AA1 was more sensitive to Janus Kinase (JAK) inhibitor JAK_8517 and AZ960_1250, insulin-like Growth Factor I Receptor (IGF1R) inhibitor IGF1R_3801, and Heat Shock Protein 90 inhibitor Luminespib_1559 (which could effectively down-regulate IGF-1R β protein). AA2 was sensitive to TGFβ/SMAD4 receptor inhibitor SB505124 **(** **Figure 5B** **)**. Moreover, using the SubMap algorithm, we also explored the correlation between the AA subtypes and response groups toward the targeted drugs cetuximab and bevacizumab. There was little significance between the AA subtypes and the bevacizumab response group, but AA1 was more relevant to the cetuximab nonresponse group (**Figure 5B**), which was consistent with the lower EGFR score in the AA1 subtype **(** **Figure 5C** **)**. Then, we compared the expression profiling of AA1 and AA2 with the melanoma dataset containing 47 patients who received a programmed cell death protein-1 (PD-1) immune checkpoint inhibitor or cytotoxic T-lymphocyte-associated protein-4 (CTLA-4) immune checkpoint inhibitor (38). AA1 exhibited a significant association with the CTLA4- and PD1-sensitive groups, which indicated that the AA1 subtype was more likely to benefit from immune checkpoint blockade therapy (**Figure 5D**).

### Generation and validation of the AA classifier in the GEO dataset

For better clinical application, we generated a classifier with a total of 60 genes containing the top 30 AAMRGs in each AA subtype **(** **Table S3** **)**. The concordance with the original prediction based on Consensus Cluster was evaluated in the TCGA cohort. We observed a concordance of 88.8% in the AA1 subtype and 89.1% in the AA2 subtype, which indicated that the 60-gene signature can reproducibly determine the COAD-AA classification. Besides, the concordance correlation coefficient (CCC) between the AA subtypes and the predicted AA subtypes was 0.78 (95% CI, 0.74-0.81). Subsequently, we validated the classifier in an independent colorectal cancer dataset, GSE41258, through the NTP algorithm. Similarly, the AA1 subclass in the GSE41258 dataset had poorer overall survival (**Figure 5E**), a higher proportion of MSI, right colon location, and worse T and N stages (**Figure 5F**).

### Exploration of the cell type-specific metabolic activity landscape with single-cell data

**Figure 6:**
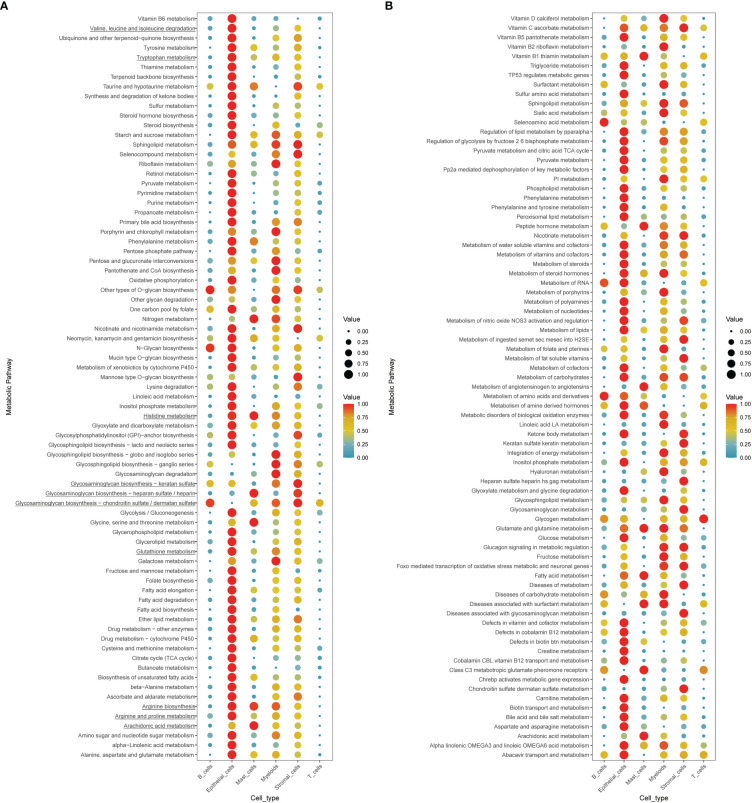
Exploration of the cell type-specific metabolic activity landscape with single-cell data. **(A, B)** Metabolic activity of different cell types in single-cell sequence data.

To further comprehend the metabolic status among different cell types in the tumor environment, we calculated the cell type-specific KEGG and REACTOME metabolic pathway activity in a single-cell RNA sequence dataset (39). As marked with underlines in the figure, we found that amino acid-related metabolic pathways, including branched-chain amino acids (BCAAs) (valine, leucine, and isoleucine), tryptophan, glutathione, and arginine metabolism, were almost highly activated in epithelial and stromal cells (**Figures 6A, B** **)**. Among immune cells, mast cells and myeloid cells acquired relatively higher amino acid metabolisms activity, such as glutamate and glutamine, arginine, and histidine metabolism. However, lymphocytes, including T cells and B cells, displayed a relatively lower metabolic level. In addition to amino acid metabolism, other metabolic processes differed in the AA subtypes like glycosaminoglycan biosynthesis and arachidonic acid metabolism, highly upregulated in the AA1 subtypes, were mainly found to be active in both stromal cells and mast cells.

## Discussion

In this study, for the first time, we established an amino acid metabolism-related gene expression-based classification to predict prognosis and assist individual treatment decisions in COAD. The two AA subtypes displayed distinct overall survival and clinical and molecular features. AA1 had an inferior overall survival and harbored ‘inactive amino acid metabolism’ and ‘inflammatory and mesenchymal TME’ characteristics. The higher MSI, TMB, and inflammatory microenvironment of AA1 contribute to its sensitivity to ICB therapy. AA2 was associated with ‘active amino acid metabolism’ features, and a higher EGFR score predicted that AA2 could benefit from EFGR inhibitor treatment.

Primary tumor location is associated with distinct differences in the microbiome, clinical characteristics, chromosomal and molecular characteristics, treatment, and prognosis (45). Left colon cancer has a better prognosis than right colon cancer under routine chemotherapy and targeted treatment. The primary locations of AA1 subtype tumors are mostly in the right colon. The right COAD is characterized by MSI, CIMP status, and BRAF/PI3KCA mutation (45) consistent with AA1 features. Meanwhile, AA1 harbors more *BRAF* mutations. The right COAD has been thought to arise through an alternative serrated neoplasia pathway (46). A sessile serrated adenoma is highlighted by *BRAF ^V600E^* point mutation, CIMP, and MLH1 methylation compared to classic adenomas (47). These results suggest that the amino acid metabolic features of right COAD and sessile serrated adenoma might be more biased toward AA1.

Next, we explored the characteristics of these two subtypes from mutation, transcriptome, tumor environment, and therapeutic response aspects. Mutations in oncogenes or tumor suppressors are known to alter cellular metabolism to fuel cancer (48). At the DNA level, there was a higher tumor mutation burden in AA1. *BRAF* and *SMAD4* are highly mutated in the AA1 subtype, and *SMAD4* mutation correlates with worse clinical outcomes and resistance to chemotherapy (49). In addition, we found high-frequency mutations, such as *TTN* (muscle protein), *SYNE1* (structural proteins), *MUC16* (O-glycosylated protein), *OBSCN* (cytoskeletal proteins), and *FAT4* (maintaining cell polarity), all of which were higher in the AA1 than AA2 type. *TTN, SYNE1, and MUC16* mutations are associated with increased TMB and correlated with an enhanced response to ICB immunotherapy in solid tumors (50–53). OBSCN mutation might promote tumor proliferation, migration, and metastasis (54, 55). *FAT4* mutation could predict survival outcomes for stratifying patients with colorectal cancer independent of TNM staging (56). These high-frequency mutations deserve further study to explore their biological functions in COAD.

Amino acids play many important roles in cell growth and survival, including participating in the TCA cycle, nucleobase synthesis, and regulating redox balance (57). Our results showed that AA2 had an extensive activation in ‘arginine and proline metabolism’, ‘tyrosine metabolism’, ‘glycine, serine, and threonine metabolism’ pathways. Arginine can be endogenously synthesized or taken up from the environment, and is critically involved in processes including the synthesis of nitric oxide and polyamines to maintain rapid proliferation (58). Due to the low expression of arginine synthesis-related enzyme, arginine auxotrophic tumors cannot endogenously synthesize sufficiently, and thus tend to depend on the uptake of extracellular arginine. Pegzilarginase, an inducer of arginine deprivation may promote an immune-stimulatory TME and improve the immunotherapy effect (59). Besides, a variety of catabolic enzymes involved in polyamine catabolism are potential targets for the treatment of COAD (58). Metabolites, rate-limiting enzymes, and ARH in tryptophan metabolism are related to CRC. Upregulated tryptophan catabolites such as kynurenine block effector T cell activation and trigger T cell apoptosis to prevent the immune system from successfully destroying cancer cells. Specifically, the enzyme IDO1breakdown tryptophan into kynurenine may be a potential therapeutic target for colorectal cancer (57). Serine and glycine contribute carbon to the serine, glycine, one-carbon (SGOC) metabolic network, which plays a role in various cellular processes, including nucleotide synthesis, lipid, and protein synthesis, methylation metabolism, polyamine metabolism, and redox balance. Serine and glycine were also reported as immunosuppressive metabolites and promoted the survival of non-transformed TME cells to form a protective niche for tumors. Hence, limiting serine metabolism may have substantial therapeutic implications for immunotherapy (60). In our results, the activation of these three metabolic sectors in AA2 is consistent with this non-beneficiary feature of immunotherapy. Therefore, we wonder whether the combination of specific metabolic inhibitors and immunotherapy can improve the response rate. Branched-chain amino acid (BCAA) metabolism, containing valine, leucine, and isoleucine, is the only upregulated amino acid metabolic pathway in the AA1 subtypes. Emerging studies have shown that the BCAA metabolic enzymes BCAT1 and BCAT2 are aberrantly activated and functionally required for malignant tumors such as chronic myeloid leukemia (61), acute myeloid leukemia (62), and PDAC (63). High levels of BCAT1 also displayed a DNA hypermethylation phenotype (62). In addition, tumor cell BCAAs and their metabolites, such as branched-chain α-keto acid, can maintain the proliferative status of Treg cells (64) or reduce the phagocytic activity of macrophages (65). BCAT1 can also downregulate glycolysis in T cells (66). Together, BCAA metabolic reprogramming plays a significant role in immune suppression, thus boosting cancer progression (67). However, clinical and biological research on BCAAs and COAD is still rare. Our work indicated that the combination of BCAA starvation or metabolic enzyme inhibitors with conventional tumor therapy might further improve the prognosis of the AA1 subtype, which needs more evidence to prove the mechanism.

Although these two subtypes did not differ significantly in age, gender, TNM stage, or AJCC stage, their prognosis was significantly different. Considering the tumor microenvironment, we hypothesized that the differences in stromal and immune-related factors contribute to the prognosis. By comparing the transcriptome data, we found that immune-, ECM- and metastasis-related pathways were significantly enriched in the AA1 subtype. ADP ribosylation, glycosaminoglycan biosynthesis, prostanoid biosynthesis, and cyclooxygenase arachidonic acid metabolism were upregulated in AA1. ADP-ribosylation (ADPR) is a posttranslational modification (68); however, its relationship with COAD survival is unknown. Glycosaminoglycan is a component of the ECM that plays an important role in supporting cells and providing a platform for cell interactions (69). Dysregulation of glycosaminoglycan metabolism, which is highly upregulated in AA1 and mainly expressed by stromal cells and mast cells, promotes sustained proliferation, angiogenesis, metastasis, and evasion of the immune response (69). PGE has been shown to promote tumor progression by silencing tumor suppressors, inducing cancer stem cell formation, enhancing immunosuppressive cells, and impairing cytotoxic CD8 T-cell and NK-cell functions (70). These dysregulated pathways in AA1 indicated that the enriched and activated stromal cells may largely contribute to the inferior prognosis of AA1. The heterogeneity of AA subtypes might also determine the different sensitivities of therapies. AA1 was more sensitive to irinotecan, ICB therapy (both anti-CTLA-4 and anti-PD-1), and bevacizumab. Meanwhile, AA1 harbored more resistance to cetuximab. Referring to the BRAF mutate COAD, the combination of anti-BRAF, anti-MEK, and anti-EGFR targeted therapy may improve the efficacy in AA1. In contrast, AA2 was more sensitive to 5-fluorouracil and oxaliplatin and may benefit more from cetuximab treatment. Finally, we established a 60-AAMRG-based classifier to simulate the 358-AAMRG classifier well, which is more concise and convenient in clinical application.

In conclusion, we built a new binary classifier of COAD, and using the AAMRG classifier can partially explain the heterogeneity of COAD. This classifier would help to timely select AA1 subtype patients who would benefit more from precise therapy and achieve better clinical outcomes.

## Data availability statement

The original contributions presented in the study are included in the article/**Supplementary Material**. Further inquiries can be directed to the corresponding authors.

## Author contributions

YX were responsible for the analysis, interpretation of data, graphing and manuscript draft. HC and J-YF supervised the whole analysis and provided guidance and instructions. All authors contributed to the article and approved the submitted version.

## Funding

This project was supported in part by grants from the National Key R&D Program of China (2020YFA0509200), and the National Natural Science Foundation of China (81830081, 31970718, 81972203).

## Conflict of interest

The authors declare that the research was conducted in the absence of any commercial or financial relationships that could be construed as a potential conflict of interest.

## Publisher’s note

All claims expressed in this article are solely those of the authors and do not necessarily represent those of their affiliated organizations, or those of the publisher, the editors and the reviewers. Any product that may be evaluated in this article, or claim that may be made by its manufacturer, is not guaranteed or endorsed by the publisher.
